# Phase I dose-escalation study of the c-Met tyrosine kinase inhibitor SAR125844 in Asian patients with advanced solid tumors, including patients with *MET*-amplified gastric cancer

**DOI:** 10.18632/oncotarget.18554

**Published:** 2017-06-16

**Authors:** Kohei Shitara, Tae Min Kim, Tomoya Yokota, Masahiro Goto, Taroh Satoh, Jin-Hee Ahn, Hyo Song Kim, Sylvie Assadourian, Corinne Gomez, Marzia Harnois, Satoshi Hamauchi, Toshihiro Kudo, Toshihido Doi, Yung-Jue Bang

**Affiliations:** ^1^ Department of Experimental Therapeutics and Gastrointestinal Oncology, National Cancer Center Hospital East, Kashiwa, Japan; ^2^ Department of Internal Medicine, Seoul National University College of Medicine, Seoul, Korea; ^3^ Division of Gastrointestinal Oncology, Shizuoka Cancer Center, Shizuoka, Japan; ^4^ Cancer Chemotherapy Center, Osaka Medical College Hospital, Osaka, Japan; ^5^ Department of Frontier Science for Cancer and Chemotherapy, Osaka University Graduate School of Medicine, Osaka, Japan; ^6^ Department of Oncology, Asan Medical Center, Seoul, Korea; ^7^ Division of Medical Oncology, Yonsei University College of Medicine, Seoul, Korea; ^8^ Research and Development, Sanofi, Paris, France; ^9^ Pharmacokinetics and Distribution, Sanofi, Paris, France

**Keywords:** gastric cancer, MET amplification, Asian population, phase I trial

## Abstract

SAR125844 is a potent and selective inhibitor of the c-Met kinase receptor. This was an open-label, phase I, multicenter, dose-escalation, and dose-expansion trial of SAR125844 in Asian patients with solid tumors, a subgroup of whom had gastric cancer and *MET* amplification (NCT01657214). SAR125844 was administered by intravenous infusion (260–570 mg/m^2^) on days 1, 8, 15, and 22 of each 28-day cycle. Objectives were to determine the maximum tolerated dose (MTD) and to evaluate SAR125844 safety and pharmacokinetic profile. Antitumor activity was also assessed. Of 38 patients enrolled (median age 64.0 years), 22 had gastric cancer, including 14 with *MET* amplification. In the dose-escalation cohort (*N* = 19; unselected population, including three patients with *MET*-amplification [two with gastric cancer and one with lung cancer]), the MTD was not reached, and the recommended dose was established at 570 mg/m^2^. Most frequent treatment-emergent adverse events (AEs) were nausea (36.8%), vomiting (34.2%), decreased appetite (28.9%), and fatigue or asthenia, constipation, and abdominal pains (each 21.1%); none appeared to be dose-dependent. Grade ≥ 3 AEs were observed in 39.5% of patients and considered drug-related in 7.9%. SAR125844 exposure increased slightly more than expected by dose proportionality; dose had no significant effect on clearance. No objective responses were observed in the dose-escalation cohort, with seven patients (three gastric cancer, two colorectal cancer, one breast cancer, and one with cancer of unknown primary origin) having stable disease. Modest antitumor activity was observed at 570 mg/m^2^ in the dose-expansion cohort, comprising patients with *MET*-amplified tumors (*N* = 19). Two gastric cancer patients had partial responses, seven patients had stable disease (six gastric cancer and one kidney cancer), and 10 patients had progressive disease. Single-agent SAR125844 administered up to 570 mg/m^2^ has acceptable tolerability and modest antitumor activity in patients with *MET*-amplified gastric cancer.

## INTRODUCTION

Interactions between the receptor tyrosine kinase mesenchymal-epithelial transition factor (c-Met), encoded by the *MET* proto-oncogene, and its ligand, the hepatocyte growth factor (HGF), trigger a broad spectrum of biologic processes involved in tumorigenesis [1].

Aberrant activation of the HGF/c-Met pathway has been observed in various solid tumors, including gastric cancer, most commonly via *MET* gene amplification. A systematic review of 15 studies in gastric cancer showed that both Asian and Western patients with a high level of c-Met have significantly poorer outcomes than do those with low levels of c-Met [2]. Other studies have similarly shown that *MET* gene amplification in gastric cancer is significantly associated with unfavorable clinical outcomes, including substantially shorter survival [3, 4]. Although the frequency of *MET* amplification in gastric cancer is generally low (2–8.3%) [3–7], targeting c-Met is a promising therapeutic approach for patients with *MET*-amplified gastric cancer.

SAR125844 (SAR) is a potent and highly selective, small-molecule c-Met kinase inhibitor. It has a half-maximal inhibitory concentration of 4.2 nM and an inhibitory constant of 2.8 nM [8]. In two xenograft models of *MET*-amplified human gastric tumors, intravenous treatment with single-agent SAR resulted in c-Met kinase inhibition and dose-dependent tumor regression. SAR also had an acceptable toxicity profile [8].

An acceptable tolerability profile was also seen in the completed first-in-human study of single-agent SAR in patients with solid tumors (NCT01391533). Preliminary evidence of antitumor activity was observed in one patient with lung adenocarcinoma and *MET* amplification [9]. That study, conducted in Western countries, explored SAR doses of 50‒740 mg/m^2^ and established the recommended dose (RD) at 570 mg/m^2^ per week, intravenously [9].

The present study (NCT01657214) has been completed and aimed to determine the maximum tolerated dose (MTD) and RD of SAR and to explore its antitumor activity in Asian patients with solid tumors, including gastric cancer, and *MET* amplification.

## RESULTS

### Demographics

In total, 38 Asian patients were treated: 19 in the dose-escalation cohort and 19 in the dose-expansion cohort. Demographics and baseline characteristics are shown in Table 1. Overall, most patients had an Eastern Cooperative Oncology Group (ECOG) performance status (PS) of 0 or 1 (92.1%), were heavily pretreated (68.4% had received three or more prior anticancer therapies), and had gastric cancer (57.9%).

**Table 1:** Patient demographics and baseline characteristics (safety population)

Characteristic	Dose-escalation cohort (*N* = 19)	Dose-expansion cohort (*N* = 19)	Total population (*N* = 38)
Age, median (range), years	60 (28–78)	65 (37–77)	64 (28–78)
Sex, *n* (%)			
Male	13 (68.4)	10 (52.6)	23 (60.5)
Female	6 (31.6)	9 (47.4)	15 (39.5)
ECOG PS, *n* (%)			
0	7 (36.8)	9 (47.4)	16 (42.1)
1	11 (58.9)	8 (42.1)	19 (50.0)
2	1 (5.3)	2 (10.5)	3 (7.9)
Primary cancer site, *n* (%)			
Stomach (gastric cancer)	8 (42.1)*	14 (73.7)	22 (57.9)
Colorectal	5 (26.3)	1 (5.3)	6 (15.8)
Lung	2 (10.5)*	2 (10.5)	4 (10.5)
Pancreas	1 (5.3)	0 (0)	1 (2.6)
Breast	1 (5.3)	0 (0)	1 (2.6)
Unknown primary	0 (0)	1 (5.3)	2 (5.3)
Kidney	0 (0)	1 (5.3)	1 (2.6)
Thymus	1 (5.3)	0 (0)	1 (2.6)
Number of previous anticancer therapies, *n* (%)			
1	0 (0)	5 (26.3)	5 (13.2)
2	4 (21.1)	3 (15.8)	7 (18.4)
≥ 3	15 (78.9)	11 (58.9)	26 (68.4)

*Two patients with gastric cancer and one patient with lung cancer from the dose-escalation cohort had *MET*-amplified tumors. In the dose-expansion cohort, all patients had *MET*-amplified tumors.

ECOG, Eastern Cooperative Oncology Group; PS: performance status.

In the dose-escalation cohort, the requirement of *MET* amplification was made optional, and only three patients (two with gastric cancer and one with lung cancer) had *MET*-amplified tumors.

In the dose-expansion cohort, a mean of 49.4% of cells had more than four gene copies of *MET* and a *MET*:*CEP7* (centromeric region of chromosome 7) ratio ≥ 2, indicating tumor *MET* amplification. Total c-Met protein expression data were available for 10 of the 19 patients from the dose-expansion cohort. Total c-Met expression was null or very low in three patients and high (> 50% of tumor cells with immunohistochemistry score of 2+ or 3+ membrane staining) in the remaining seven patients. Overall, mean level of total c-Met expression was 56%. No correlation was found between total c-Met expression and *MET* amplification (Supplementary Table 1). Of the approximately 400 patients who underwent prescreening, *MET* amplification was found in 73, of whom 19 were treated in the dose-expansion cohort. Of these, 14 (73.7%) had gastric cancer, one (5.3%) had colorectal cancer, and two (10.5%) had lung cancer. Compared with other tumors, gastric cancer tumors had higher percentages of cells with > 4 copies of *MET* and *MET*:*CEP7* ≥ 2.

### Treatment exposure

The median duration of treatment was 4.1 (range 2−38) weeks. Details of the number of infusions and treatment duration per cohort are indicated in Supplementary Table 2.

### MTD and RD

No dose-limiting toxicities (DLTs) were observed during the dose-escalation phase. The MTD was not reached, and 570 mg/m^2^ was selected as the RD in Asian patients because of similar exposure and safety profile to those seen in Western patients [9]. Of 19 patients treated in the expansion cohort at 570 mg/m^2^, one experienced a DLT (transaminase and creatinine increases that were reversible after dose omission and reduction) during cycle 1.

### Safety

Treatment-emergent adverse events (TEAEs) were observed in 36 patients (94.7%) and considered study-drug related in 22 patients (57.9%). Serious TEAEs were reported in eight patients (21.1%); none were considered study-drug related. The most frequent TEAEs (Table 2) were nausea (36.8%), vomiting (34.2%), decreased appetite (28.9%), and fatigue or asthenia, constipation, and abdominal pains (21.1% each). The frequency and severity of TEAEs at each dose were comparable. Grade ≥ 3 TEAEs were observed in 15 patients (39.5%) and considered study-drug related in three patients. There were no deaths due to adverse events. Nine patients had at least one dose modification, and one patient had an infusion interrupted because of a grade 2 infusion-related reaction. One patient discontinued treatment because of a TEAE considered unrelated to the study drug. No major safety concerns were raised by laboratory findings. Two cases of transaminase increase (grade ≥ 3) led to dose modification. No creatinine increases were observed. Anemia was mainly observed in patients with gastric cancer and was attributed to the primary cancer.

**Table 2:** Summary of the most common treatment-emergent adverse events occurring in ≥ 10% of all patients

TEAE, *n* (%)	Dose-escalation cohort Dose, mg/m^2^				Dose-expansion cohort		Total (*N* = 38)
	260 (*N* = 6)	340 (*N* = 4)	440 (*N* = 3)	570 (*N* = 6)	All (*N* = 19)	GC (*N* = 14)	
Nausea	1 (16.7)	2 (50)	0 (0)	3 (50)	8 (42.1)	4 (28.6)	14 (36.8)
Vomiting	1 (16.7)	1 (25.0)	0 (0)	1 (16.7)	10 (52.6)	6 (42.9)	13 (34.2)
Decreased appetite	1 (16.7)	1 (25.0)	1 (33.3)	0 (0)	8 (42.1)	5 (35.7)	11 (28.9)
Constipation	2 (33.3)	0 (0)	1 (33.3)	1 (16.7)	4 (21.1)	2 (14.3)	8 (21.1)
Fatigue/asthenia	3 (50)	0 (0)	1 (33.3)	0 (0)	4 (21.1)	2 (14.3)	8 (21.1)
Abdominal pains	1 (16.7)	0 (0)	1 (33.3)	0 (0)	6 (31.5)	2 (14.3)	8 (21.1)
Pyrexia	4 (66.7)	0 (0)	0 (0)	0 (0)	3 (15.8)	1 (7.1)	7 (18.4)
Injection site reaction/phlebitis	1 (16.7)	0 (0)	0 (0)	1 (16.7)	5 (26.3)	4 (28.6)	7 (18.4)
Back pain	3 (50.0)	0 (0)	0 (0)	0 (0)	2 (10.5)	2 (14.3)	5 (13.2)
Diarrhea	0 (0)	0 (0)	0 (0)	0 (0)	5 (26.3)	4 (28.6)	5 (13.2)
Cancer pain	0 (0)	0 (0)	1 (33.3)	2 (33.3)	1 (5.3)	1 (7.1)	4 (10.5)
Edema, peripheral	0 (0)	0 (0)	0 (0)	1 (16.7)	3 (15.8)	2 (14.3)	4 (10.5)
Insomnia	1 (16.7)	0 (0)	0 (0)	0 (0)	3 (15.8)	2 (14.3)	4 (10.5)
Rash	2 (33.3)	1 (25.0)	0 (0)	0 (0)	1 (5.3)	1 (7.1)	4 (10.5)
Cough	2 (33.3)	1 (25.0)	0 (0)	0 (0)	1 (5.3)	1 (7.1)	4 (10.5)

GC, gastric cancer; TEAE, treatment-emergent adverse events.

### Pharmacokinetics

**Figure 1:**
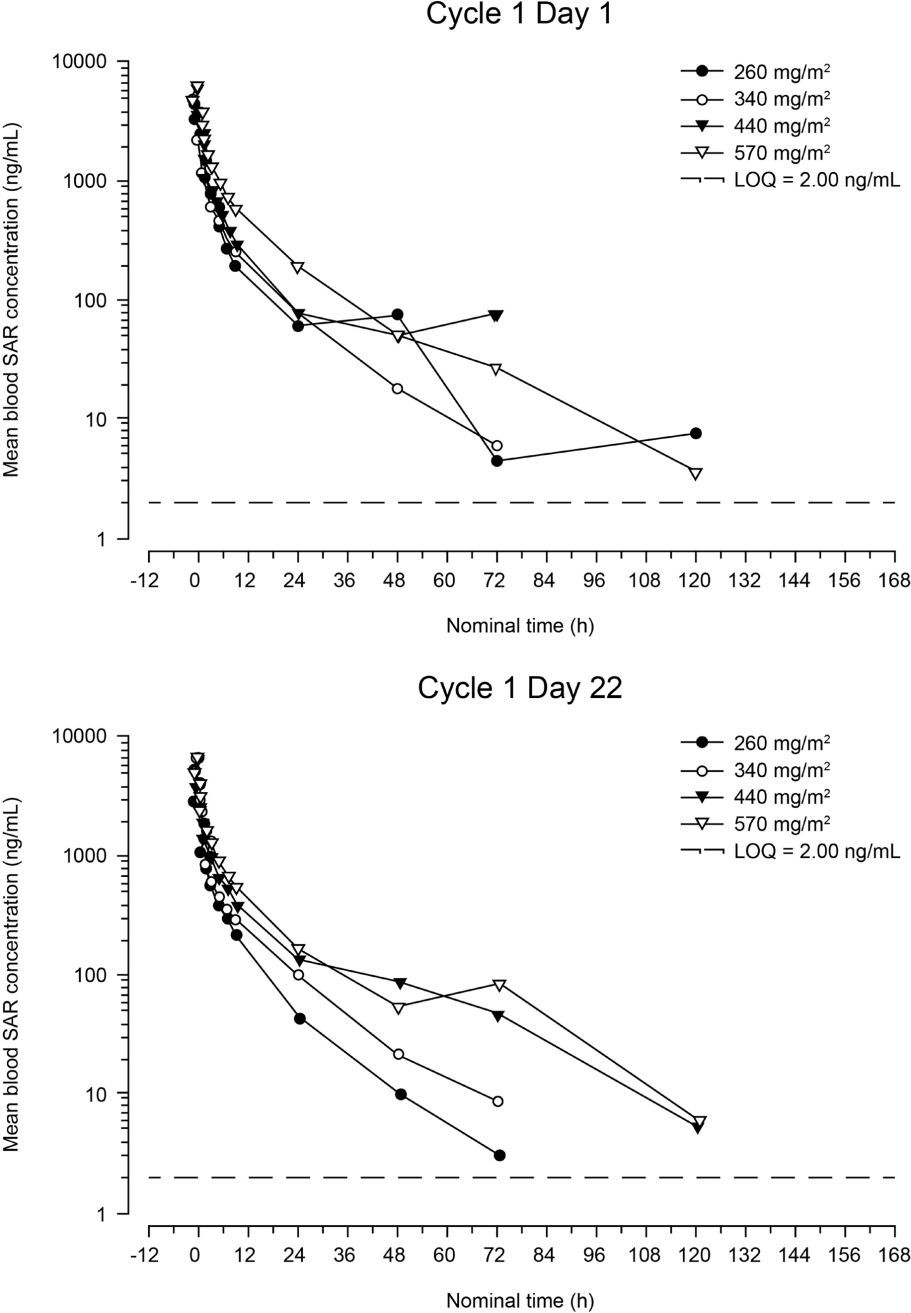
Mean blood concentration of SAR versus time after first infusion (cycle 1, day 1) and fourth infusion (cycle 1, day 22) (semi-logarithmic scale) LOQ = lower limit of quantification.

Profiles of the mean blood concentration of SAR over time on days 1 and 22 of cycle 1 are presented in Figure 1. Pharmacokinetic parameters are shown in Table 3. SAR exposure (area under the concentration-time curve for the period covering the dosing interval [AUC_0–168_]) increased slightly more than dose proportionally over the 260- to 570-mg/m^2^ dose range: a 2.19-fold increase in dose resulted in a 3.00- and a 2.69-fold increase in AUC_0–168_, on days 1 and 22, respectively. This finding is reinforced by the results of the statistical analysis, which showed no significant effect of dose on total body clearance (CL; *P* = 0.25) or CL at steady state (CL_s_; *P* = 0.79). Pharmacokinetic parameters were similar on days 1 and 22, and no accumulation was observed between days 1 and 22. SAR exhibited a large volume of distribution at steady state (approximately 253 L at the RD).

**Table 3:** Pharmacokinetic parameters after single (cycle 1, day 1) or repeated administration (cycle 1, day 22) of SAR, presented as mean ± standard deviation (geometric mean) [coefficient of variation, %]

	SAR dose in cycle 1							
	260 mg/m^2^		340 mg/m^2^		440 mg/m^2^		570 mg/m^2^	
	Day 1 *N* = 6	Day 22 *N* = 2	Day 1 *N* = 2	Day 22 *N* = 3	Day 1 *N* = 3	Day 22 *N* = 3	Day 1 *N* = 21	Day 22 *N* = 17
C_max_, ng/mL	4140 ± 1070 (4040) [26]	3550 ± NC (3540) [NC]	4290 ± NC (4280) [NC]	4700 ± 976 (4640) [21]	6390 ± 1010 (6330) [16]	5840 ± 1420 (5710) [24]	6570 ± 1680 (6390) [26]	6330 ± 1270 (6210) [20]
AUC_0–168_, ng.h/mL	11800 ± 2960 (11500) [25]^a^	12700 ± NC (12700) [NC]	16400 ± NC (16400) [NC]	16500 ± 2390 (16400) [14]	22100 ± NC (21800) [NC]^b^	25300 ± 5220 (25000) [21]	36100 ± 11800 (34400) [33]^c^	35100 ± 14900 (33000) [43]
AUC, ng.h/mL	11800 ± 2950 (11500) [25]^a^	12600 ± NC (12600) [NC]	16400 ± NC (16400) [NC]	16500 ± 2390 (16400) [14]	22000 ± NC (21800) [NC]^b^	25400 ± 5300 (25100) [21]	36200 ± 11800 (34400) [33]^c^	35100 ± 15000 (33100) [43]
CL, L/h	36.5 ± 7.68 (35.8) [21]^a^	32.0 ± NC (32.0) [NC]	33.9 ± NC (33.3) [NC]	33.4 ± 8.01 (32.8) [24]	31.8 ± NC (31.6) [NC]^b^	30.2 ± 7.54 (29.5) [25]	27.8 ± 8.94 (26.3) [32]^c^	28.9 ± 8.48 (27.6) [29]
V_ss_, L	232 ± 57.5 (227) [25]^a^	186 ± NC (186) [NC]	246 ± NC (242) [NC]	291 ± 52.6 (288) [18]	202 ± NC (200) [NC]^b^	490 ± 366 (413) [75]	241 ± 55.3 (235) [23]^c^	287 ± 105 (271) [37]
t_1/2z_, h	12.6 ± 6.05 (11.7) [48]^a^	12.6 ± NC (12.6) [NC]	12.9 ± NC (12.9) [NC]	20.6 ± 4.69 (20.2) [23]	12.1 ± NC (12.1) [NC]^b^	27.5 ± 8.24 (26.8) [30]	22.8 ± 9.47 (21.0) [42]^c^	21.2 ± 4.94 (20.6) [23]

^a^*n* = 5 (one patient not included in calculation of summary statistics); t_1/2z_ not calculable.

^b^*n* = 2 (one patient not included in calculation of summary statistics); t_1/2z_ not calculable.

^c^*n* = 20 (one patient not included in calculation of summary statistics); t_1/2z_ not calculable.

Cycle 1, day 1: Six patients were excluded from descriptive statistics. Cycle 1, day 22: Five patients were excluded from descriptive statistics. NC, not calculated.

### Antitumor activity

No objective responses were observed in the dose-escalation cohort (unselected population). Although 7 patients (three with gastric cancer, two with colorectal cancer, one with breast cancer, and one with cancer of unknown primary origin) had stable disease after 1 month of treatment, all seven stopped treatment within the subsequent 2 months because of disease progression; 11 patients had early disease progression leading to treatment discontinuation in cycle 1. Best overall response and median duration of treatment by dose cohort are shown in Supplementary Figure 1. Median time to tumor progression ranged from 0.9 months at 340 mg/m^2^ to 1.7 months at 440 mg/m^2^.

**Figure 2:**
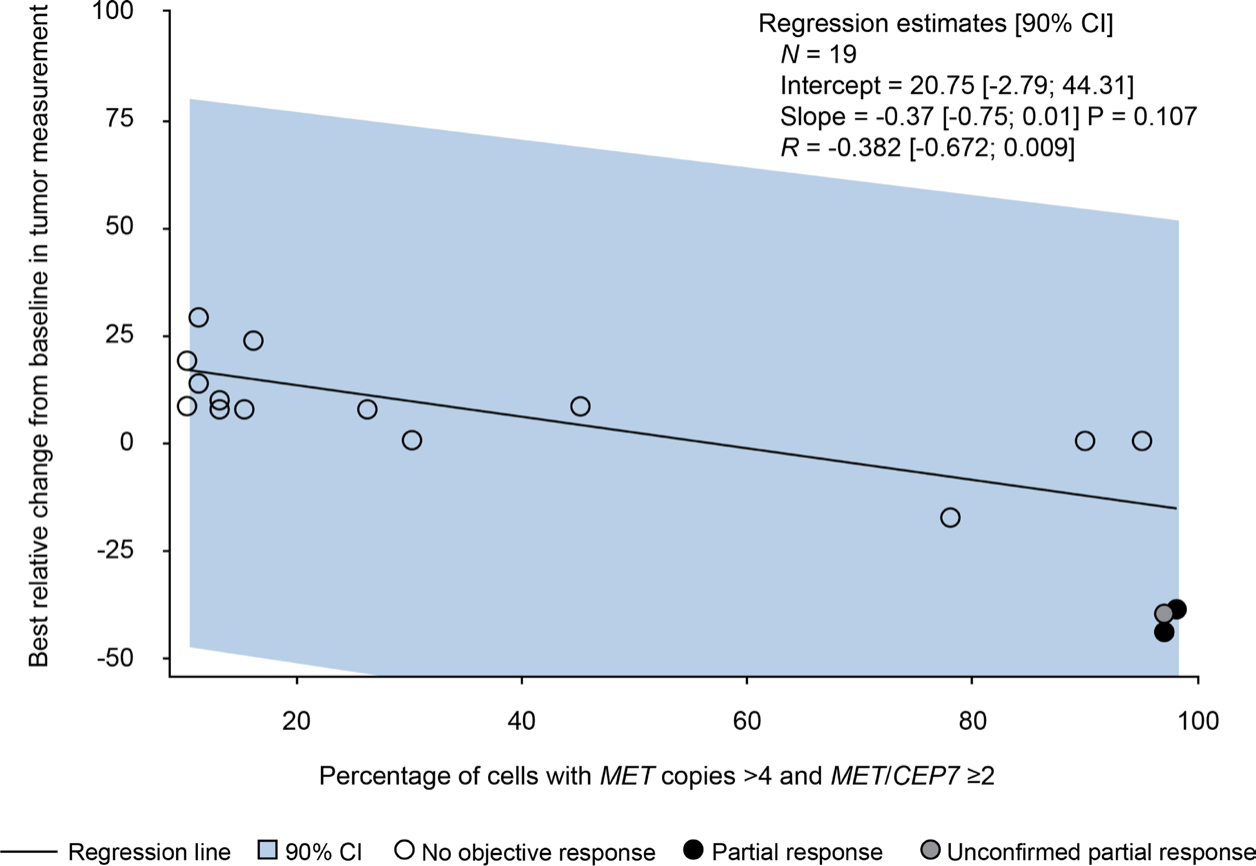
Best relative change from baseline in tumor measurement and level of *MET* amplification (from fluorescence *in situ* hybridization results) CI = confidence interval.

In the selected population with *MET*-amplified tumors (dose-expansion cohort), two patients (10.5%), both with gastric cancer, had a confirmed partial response, and one patient (also with gastric cancer) had an unconfirmed partial response. These three responders had high levels of *MET* amplification (≥ 97% amplified cells). However, given the small sample number, no correlation could be identified between the level of *MET* amplification and best change from baseline in tumor measurement (Figure 2). Overall, within the dose-expansion cohort, seven patients (six with gastric cancer and one with kidney cancer) had stable disease and 10 patients (six with gastric cancer, two with lung cancer, one with colorectal cancer, and one with cancer of unknown primary origin) had progressive disease. Best overall response and median duration of treatment are shown in Supplementary Figure 1. Median time to tumor progression was 0.92 (0.4–9) months. In the gastric cancer subpopulation, overall response rate was 14.3% (two of 14 patients) and median time to tumor progression was 1.18 (0.4–9) months.

## DISCUSSION

This study aimed to establish the RD of a weekly administration of SAR in Asian patients with solid tumors. No DLTs were observed in the dose-escalation phase, and 570 mg/m^2^ was selected as the RD based on comparable exposure and safety in Asian and Western populations [9]. SAR had an acceptable safety profile, with no major safety concerns and no relevant dose-dependent AEs. Most frequent TEAEs were nausea, vomiting, decreased appetite, fatigue or asthenia, constipation, and abdominal pains. Similar AEs have been reported with other c-Met inhibitors [10–12] and may represent a class effect. SAR exposure levels were slightly higher than predicted by dose proportionality, but no accumulation was seen after four repeated once-weekly infusions, and clearance was medium. At the RD, SAR had a large volume of distribution at steady state, and the elimination half-life was approximately 21 hours. Modest antitumor activity was observed: the objective response rate was 10.5% (two of 19) in patients with *MET*-amplified solid tumors; two gastric cancer patients had a partial response and one gastric cancer patient had an unconfirmed partial response. The partial response rate was 14.3% (two of 14) in gastric cancer patients with *MET* amplification. Notably, this preliminary efficacy was observed in tumors that had a high level (≥ 97%) of *MET* amplification.

Studies of other c-Met inhibitors in gastric cancer have shown a variety of efficacy outcomes, and differences are probably related to population heterogeneity and the definition of *MET* gene amplification. Trials of tivantinib, a c-Met inhibitor, and foretinib, a c-Met/vascular endothelial growth factor receptor 2 inhibitor, in unselected gastric cancer patients have shown no objective responses [13, 14]. However, treatment with the multikinase inhibitor crizotinib led to tumor shrinkage in two of four patients with highly *MET*-amplified (*MET*:*CEP7* >5) gastroesophageal cancers [15]. Treatment with AMG 337, a selective c-Met kinase inhibitor, led to a partial or near complete response in eight (62%) of 13 patients with *MET*-amplified gastroesophageal junction, gastric, or esophageal tumors [16]. All studies used local fluorescence *in situ* hybridization (FISH) to identify whether a tumor was *MET* amplified or not. However, the cut-off for defining *MET* gene amplification differed. The studies of tivantinib and crizotinib considered tumors with a ratio of *MET:CEP7* ≥ 2.2 as *MET* amplified [13, 15], whereas the foretinib study used the cut-off of *MET:CEP7* ≥ 2 to define *MET* amplification [14]. In the study with AMG 337, the cut-off value for defining *MET* amplification is not provided [16].

Combination therapy of c-Met-targeted antibodies and first-line chemotherapeutics in gastric cancer has shown no clear clinical benefit. For example, addition of onartuzumab (an anti-c-Met antibody) to chemotherapy provided no survival advantage, regardless of c-Met expression [17], and rilotumumab (an anti-HGF antibody) combined with chemotherapy failed to show clinical benefit in c-Met-positive gastric or gastroesophageal junction cancer [18].

Preclinical studies provide a wealth of data demonstrating antitumor activity of small-molecule c-Met kinase inhibitors [19–22]. In the clinical setting, our study and some others indicated preliminary efficacy in gastric cancer patients with *MET* amplification [15, 16]. However, there are several challenges to fully demonstrating the clinical benefit of these agents. The low frequency of *MET* amplification in gastric cancer is a barrier to the inclusion of large-enough numbers of patients in trials. Also, stringent methods for assessing amplification must be used. A recent study using next-generation sequencing revealed that amplification of receptor tyrosine kinases does not correlate with protein expression in gastric cancer, and a comprehensive analysis using both next-generation sequencing and immunohistochemistry may be necessary to adequately select patients [7]. Additional challenges to assess efficacy of c-Met are the definition of gene amplification and intratumoral heterogeneity. Future strategies for c-Met kinase receptor inhibitors evaluation in gastric cancer will require stringent and standardized methods to assess *MET* amplification, will likely use a high threshold for the definition of amplification [23], and will consider amplification clonality in patient selection. A marked heterogeneity of *MET* amplification in distinct tumor lesions within a single gastric cancer patient has been reported, and this heterogeneity may be a key driver of resistance to c-Met inhibitors [24].

## MATERIALS AND METHODS

### Patient population

Asian patients were recruited from multiple centers. Key eligibility criteria were age ≥ 20 years, a solid tumor for which no standard therapy was available (dose-escalation phase) plus measurable disease and *MET* gene amplification (dose-expansion phase), ECOG PS of 0–2, and adequate hematologic, hepatic, and renal function. Exclusion criteria included previous treatment with any c-Met inhibitor, unresolved grade > 1 toxicity (excluding alopecia) related to any prior anticancer therapy, a washout period of < 3 weeks since previous antitumor therapy or investigational treatment (or < 6 weeks if the previous treatment was nitrosourea or mitomycin C), or known hypersensitivity/experienced any AE related to the study-drug excipient Captisol^®^ (Ligand Pharmaceuticals, Lawrence, Kansas, US). All patients provided written informed consent.

### Study design and treatment

This was an open-label, phase I, multicenter study consisting of a dose-escalation phase (to determine the MTD of SAR) and a dose-expansion phase (to evaluate the antitumor activity of SAR at the RD in patients with *MET*-amplified tumors, including gastric cancer).

Dose escalation was guided by a Bayesian logistic regression model, an adaptive model with overdose control used to estimate whether the probability of a DLT at each candidate dose level is within a targeted interval of 20‒35%. Dose escalation was indicated if the probability of a DLT within the targeted interval at the next level was greater than at the current level, if the risk of overdosing (DLT rate > 35%) was controlled (i.e., below a pre-specified 25% level). Dose escalation did not proceed until at least three patients had been treated at the lower dose level. The model was run based on DLTs occurring during the first cycle of treatment.

SAR was administered by weekly intravenous infusion on days 1, 8, 15, and 22 of each 28–day cycle. The starting dose was 260 mg/m^2^, and dose increments of 30% were made, to a maximum of 570 mg/m^2^. Treatment continued until disease progression, unacceptable toxicity, or consent withdrawal. Patients were screened within 21 days before the first administration and followed for 30 days after the last administration.

The study was approved by the institutional review board or independent ethics committee of each participating center, and was conducted in accordance with the Declaration of Helsinki and the International Council for Harmonisation Guideline for Good Clinical Practice.

### Assessments

The MTD was defined as the dose having the highest probability of generating 20‒35% of study drug-related DLTs, and was primarily determined based on DLTs observed in cycle 1. A DLT was defined as grade 4 neutropenia for ≥ 7 consecutive days, febrile neutropenia, grade 4 thrombocytopenia or grade 3 thrombocytopenia with bleeding requiring a transfusion, any grade ≥ 3 nonhematologic event not easily managed or corrected by medical intervention, or SAR-related toxicity resulting in two or more missed doses.

In the absence of DLTs at the highest administered dose, relevant information from the first-in-human study [9] was considered in dose-escalation decisions and determination of the MTD and RD. Safety assessments were conducted at regular intervals, and AEs were graded per the National Cancer Institute’s Common Terminology Criteria for Adverse Events, version 4.03. The safety population consisted of all patients exposed to SAR, regardless of the amount of treatment administered.

Blood samples for pharmacokinetic analysis were acquired using dried blood-spot technology on days 1 and 22 of cycle 1 at several time points before, during, and after infusion (up to 168 hours after administration). Drug concentrations were determined by a validated method of liquid chromatography and tandem mass spectrometry (limit of quantification 2 ng/mL). Pharmacokinetic parameters were calculated using a noncompartmental analysis and included the maximum observed blood concentration (C_max_), AUC from 0 to infinity, AUC_0–168_, CL, CL after repeated administrations (CL_ss_), volume of distribution at steady state (V_ss_), and the half-life associated with the terminal slope (t_1/2z_).

Tumor response was evaluated by the Response Evaluation Criteria In Solid Tumors version 1.1, which define complete response as disappearance of all target lesions, partial response and progressive disease as a minimally 30% decrease or 20% increase, respectively, in the sum of diameters of target lesions, and stable disease as neither sufficient shrinkage to qualify for partial response nor sufficient increase to qualify for progressive disease [25]. Tumor burden was assessed from computerized tomography or magnetic resonance imaging scans performed at baseline, at the end of cycle 1, and every 8 weeks thereafter. Patients evaluable for efficacy received at least one cycle of SAR, with a minimum of two infusions, and had undergone efficacy assessment at baseline and once or more after that.

In the dose-expansion cohort, *MET* amplification in tumor tissue was evaluated by a FISH assay. A tumor was considered *MET* amplified if ≥ 10% of cells had more than four gene copies of *MET* and the *MET*:*CEP7* ratio was ≥ 2. Total c-MET protein expression was evaluated by immunohistochemistry, calculating the percentage of tumor cells with an immunohistochemistry score of 2+ or 3+ membrane staining. Level of c-Met expression was considered high if > 50% of tumor cells had an immunohistochemistry score of 2+ or 3+ membrane staining. Immunohistochemistry was performed on fresh or archival tumor tissue.

### Sample sizes and statistical methods

It was estimated that 25–45 patients would be needed to establish the MTD and RD of SAR. A maximum of 25 patients (including 15 with gastric cancer) were to be treated at the MTD (expansion cohort). Descriptive statistics were used to summarize patient characteristics, safety, exposure, and antitumor activity.

## SUPPLEMENTARY MATERIALS FIGURE AND TABLES



## Abbreviations

AEs: adverse events
AUC_0–168_: area under the concentration-time curve for the period covering the dosing interval
CEP7: centromeric region of chromosome 7
c-Met: tyrosine protein kinase Met
CI: confidence interval
CL: total body clearance
CL_ss_: total body clearance at steady state
C_max_: maximum observed blood concentration
DLT: dose-limiting toxicity
ECOG: Eastern Cooperative Oncology Group
LOQ: lower limit of quantification
MET: mesenchymal-epithelial transition factor gene
MTD: maximum tolerated dose
PS: performance status
SAR125844: SAR
t_1/2z_: the half-life associated with the terminal slope
TEAE: treatment-emergent adverse event
RD: recommended dose
V_ss_: volume of distribution at steady state
