# Supplementary material for: Phase I dose-escalation study of the c-Met tyrosine kinase inhibitor SAR125844 in Asian patients with advanced solid tumors, including patients with MET-amplified gastric cancer
Source: Oncotarget. 2017 Jun 16;8(45):79546–55. doi: 10.18632/oncotarget.18554 (PMC5668067; doi:10.18632/oncotarget.18554)
Supplement: Supplementary file 1 [file oncotarget-08-79546-s001.pdf]

Phase I dose-escalation study of the c-Met tyrosine kinase inhibitor SAR125844 in Asian patients with advanced solid tumors, including patients with *MET*-amplified gastric cancer

SUPPLEMENTARY MATERIALS

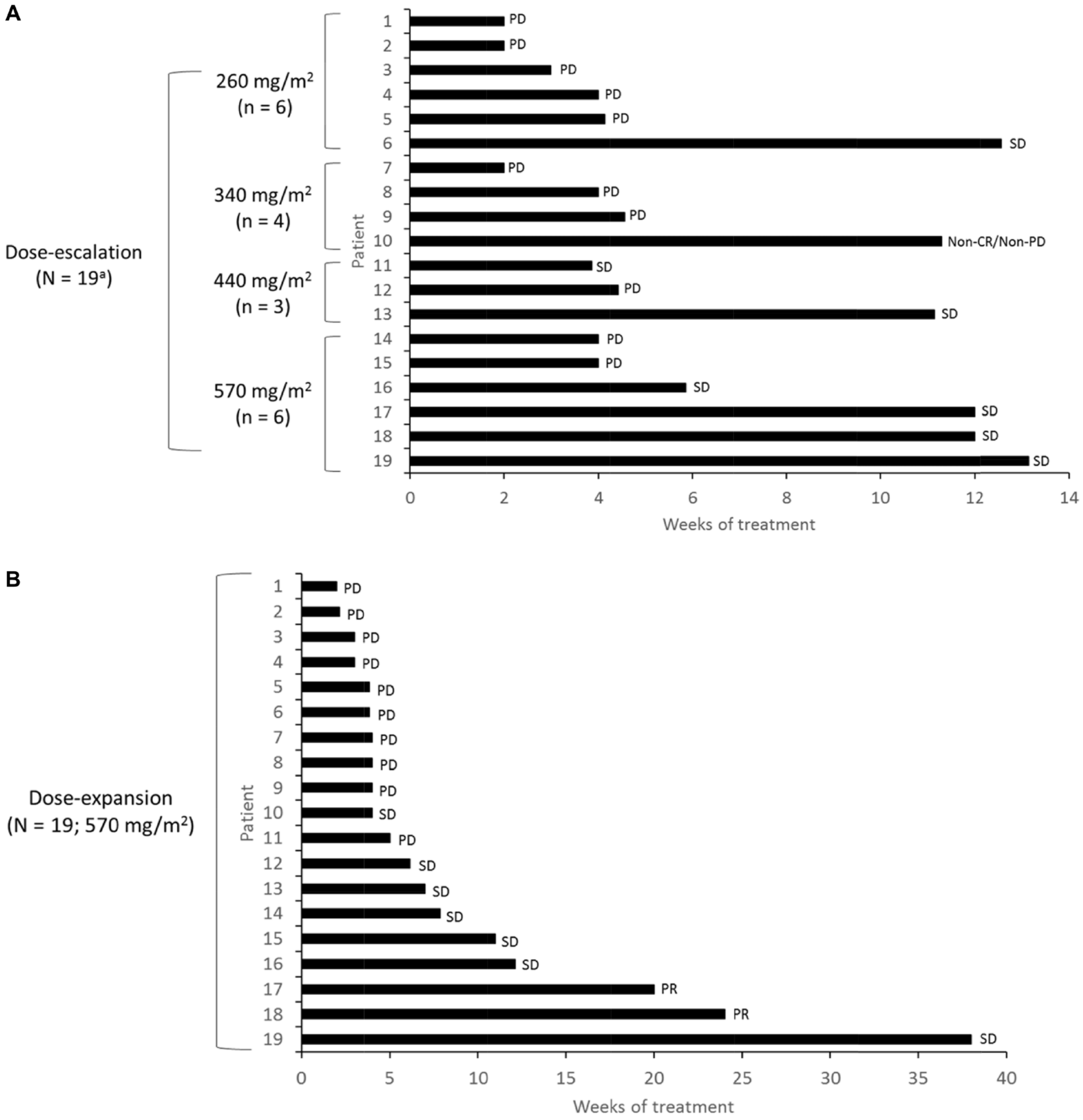

Supplementary Figure 1: Duration of treatment and best response for each patient in the (**A**) dose-escalation and (**B**) dose-expansion phases. [a]One patient in dose cohort 340 mg/m^2 only had nontarget lesions at baseline. CR = complete response; PD = progression of disease; PR = partial response; SD = stable disease.

Supplementary Table 1: Total c-Met protein expression (IHC) and respective *MET* amplification in patients in the dose-expansion cohort ($n = 10$)

Patient	Total c-Met (percentage of cells with 2+ or 3+ intensity)*	*MET* amplification (%)**
1	93	11
2	75	26
3	85	16
4	93	90
5	0	10
6	50	78
7	1	45
8	95	86
9	0	11
10	85	95

*cMet overexpression is defined when there are ≥ 50% of tumor cells with 2+ or 3+ positive membrane stain on immunohistochemistry (IHC).
**MET* amplification is defined when there are ≥ 10% of cells with > 4 *MET* gene copies, and a cMET/CEP7 ratio ≥ 2 as determined by fluorescence *in situ* hybridization.

Supplementary Table 2: Summary of study treatment exposure (safety population)

	Dose in dose-escalation cohort, mg/m^2				Dose-expansion cohort	
	260 ($N = 6$)	340 ($N = 4$)	440 ($N = 3$)	570 ($N = 6$)	All patients ($N = 19$)	Gastric cancer ($N = 14$)
Median treatment duration (range), weeks	3.5 (2−13)	4.3 (2−11)	4.4 (4−11)	8.9 (4−13)	4.0 (2−38)	5.6 (2−38)
Total number of infusions administered (all patients), *n*	27	21	19	49	157	133
Total number of infusions administered per patient, median *n* (range)	3.5 (2−12)	4 (2−11)	4 (4−11)	8.5 (4−12)	4 (2−38)	5.5 (2−38)
